# Liver Transplantation for Colorectal Metastases: Impact of a Standardised Protocol for Patient Selection on Transplant Outcomes

**DOI:** 10.3390/cancers17122046

**Published:** 2025-06-19

**Authors:** Alberto Stocco, Andrea Laurenzi, Matteo Serenari, Enrico Prosperi, Guido Fallani, Chiara Bonatti, Giorgia Radi, Margherita Prior, Federica Odaldi, Chiara Zanfi, Federica Mirici Cappa, Antonio Siniscalchi, Maria Cristina Morelli, Matteo Ravaioli, Matteo Cescon

**Affiliations:** 1 Hepatobiliary and Transplant Surgery Unit, IRCCS Azienda Ospedaliero-Universitaria di Bologna, Via Massarenti, 9, 40138 Bologna, Italy; alberto.stocco3@unibo.it (A.S.); andrea.laurenzi@aosp.bo.it (A.L.); matteo.ravaioli6@unibo.it (M.R.); 2 Department of Medical and Surgical Sciences (DIMEC), University of Bologna, 40138 Bologna, Italy; 3 Internal Medicine Unit for the Treatment of Severe Organ Failure, IRCCS Azienda Ospedaliero-Universitaria di Bologna, 40138 Bologna, Italy; 4 Postoperative and Abdominal Organ Transplant Intensive Care Unit, IRCCS Azienda Ospedaliero-Universitaria di Bologna, 40138 Bologna, Italy

**Keywords:** liver transplantation, colorectal liver metastases, transplant oncology, recurrence patterns, liver multidisciplinary evaluation

## Abstract

**Background:** Colorectal liver metastases (CRLM) occur in up to 50% of colorectal cancer with a significant impact on patient survival, of whom only 20–30% will be considered suitable for surgical treatment. Despite the progress in systemic therapies, palliative chemotherapy alone results in a 5-year overall survival (OS) < 10%. Recently, liver transplantation (LT) has been reconsidered as an option and demonstrates improved survival in highly selected patients. This study assessed the impact of implementing a standardised patient selection protocol (LITORALE) on post-transplant outcomes for unresectable CRLM (uCRLM) at a high-volume single centre. Methods: This is a prospective observational study including all consecutive patients transplanted for uCRLM at our institution between July 2015 and September 2024. This prospective observational study evaluated the impact of the LITORALE protocol on post-transplant outcomes in uCRLM patients at a single centre. Patients who underwent LT between July 2015 and September 2024 were grouped into pre-LITORALE (2015–2021) and LITORALE (post-2021) cohorts. Recipient profiles, transplant variables, and post-transplant outcomes were compared. **Results**: Twenty-one patients were included (eight pre-LITORALE, thirteen LITORALE). The LITORALE group had a lower median number of lesions (4 vs. 17.5, *p* = 0.004), a smaller major lesion size (3 cm vs. 5.5 cm, *p* = 0.082), and a significantly lower tumour burden score (6.32 vs. 18.02, *p* = 0.002). Similar to recent major clinical trials, one- and three-years OS were 100% and 83%, respectively, after protocol introduction; recurrence patterns were significantly different, with reduced multi-site recurrences (7.7% vs. 50%, *p* = 0.048) and a higher incidence of lung-only recurrences in the LITORALE group (50% vs. 0%, *p* = 0.033). **Conclusions**: The introduction of the LITORALE protocol significantly influenced patient selection and recurrence patterns in LT for uCRLM. Although the limited number of patients and the short study timespan highlight the need for future validation, these preliminary results support the adoption of structured, multidisciplinary criteria to optimise oncologic outcomes.

## Simple Summary

Despite significant advances in cancer care, patients with unresectable colorectal liver metastases still face a poor prognosis. Recent research suggests that liver transplantation could offer a second chance, but only for patients who meet very specific criteria. At our centre, we introduced a standardised protocol called “LITORALE” to better identify suitable candidates for transplantation. This study compared outcomes before and after applying the protocol. We found that patients selected with LITORALE had fewer and smaller tumours and showed more favourable recurrence patterns, such as fewer widespread relapses and more cases limited to the lungs, which are easier to manage. These preliminary results support the use of strict and multidisciplinary selection criteria to improve outcomes in this complex patient population and may help guide future strategies in liver transplantation for colorectal liver metastases.

## 1. Introduction

Colorectal cancer (CRC) is the third most frequent malignancy worldwide, and it is one of the leading causes of cancer-related death [1,2,3] Approximately 50% of CRC patients will develop liver metastases (CRLM), of whom only 20–30% will be considered suitable for surgical treatment, which represents the gold standard to achieve cure [4]. The majority of patients with CRLM are not eligible for surgery due to anatomical, functional, or oncological limitations; for those patients, palliative chemotherapy remains the sole therapeutic possibility, with a 10% expected 5-year overall survival (OS) [5,6].

In the past, liver transplantation (LT) was considered an unsuitable treatment for CRLM because of the poor survival outcomes shown by preliminary procedures in the 80-90ss [7]. Over the years—with the experience accumulated in patients transplanted for other oncological indications (hepatocellular carcinoma, perihilar cholangiocarcinoma, neuroendocrine metastases), and with the improvements in perioperative management, immunosuppression protocols, and chemotherapeutic treatment—LT for unresectable colorectal liver metastases (uCRLM) has regained popularity amongst clinicians [7,8].

The 2013 Scandinavian SECA I trial first showed satisfactory survival outcomes in patients transplanted for uCRLM [9], and the subsequent SECA II trial demonstrated a further improvement in overall survival and disease-free survival introducing more restrictive criteria for patient selection, accounting also for tumour biology [10]. In 2024, LT was demonstrated to have significant survival advantage compared to sole chemotherapy in the TransMet randomised controlled trial, which showed a 56.6% overall 5-year survival in the intention-to-treat population compared with only 12.6% of chemotherapeutic treatment alone. This trial posed critical emphasis on multidisciplinary tumour board patient assessment, which evaluated unresectability, response to therapy, and absence of extrahepatic disease with the aim of standardizing eligibility criteria for transplant [11]. Transplant eligibility was further refined in a recent prospective study from the University of Rochester Medical Center, which showed excellent outcomes after living-donor liver transplantation (LDLT) applying strict tumour biology selection criteria [12]. Currently, several randomised and prospective trials are investigating the results of LT for uCRLM, whose results are expected to provide further evidence on optimal selection criteria and also to advocate for a revision of transplant allocation policies [13,14].

The objective of this study is to investigate post-operative and survival outcomes of LT for CRLM at our institution, comparing patients transplanted before and after the introduction of a standardised selection criteria protocol (Liver Transplantation for Non-Resectable Liver Metastasis, LITORALE).

## 2. Methods

This is a prospective observational study conducted at the Hepatobiliary Surgery and Transplantation Unit of Policlinico Sant’Orsola, IRCCS Azienda Ospedaliero-Universitaria di Bologna. The study enrolled all consecutive patients transplanted for uCRLM at our institution between July 2015 and September 2024. Study population was divided into two groups according to the introduction of our standardised protocol (LITORALE2020, NCT05185245) in April 2021: pre-LITORALE (2015–2021) and LITORALE (after 2021).

### 2.1. LITORALE Inclusion Criteria

After 2021, patients were considered eligible for transplant with an age between 18 and 73 years, good performance status (ECOG 0-1) and a confirmed diagnosis of metastatic adenocarcinoma of the colon or rectum ≤ pT4a, whose primary tumour had undergone previous curative intent radical surgery (R0). Metastases could be considered unresectable either at the time of initial diagnosis or after initial liver surgery, without evidence of local recurrence of the primary tumour or extrahepatic disease (assessed by positron emission tomography (PET), computed tomography, and colonoscopy). Further eligibility criteria included a neutrophil count greater than 1.0 × 10^9^/L (without need for granulocyte colony-stimulating factor administration) and stable disease (SD) or partial response (PR), as per mRECIST criteria, after a minimum of one cycle of chemotherapy for at least three months. Additionally, carcinoembryonic antigen (CEA) < 80 μg/L (or a 50% CEA reduction from the highest recorded level) and absence of transplant contraindications were required. Patient’s eligibility for protocol inclusion was based on mandatory evaluation in a multidisciplinary board which included oncologists, hepatologists, surgeons with proved experience in liver resection and transplantation (MC and MR), radiologists, and pathologists. For patients referred to our centre for evaluation for LT by other centres, metastases had already been judged unresectable in those centres, with or without a history of previous liver resection(s).

Exclusion criteria included history of other malignancies, local recurrence of the primary tumour, extrahepatic disease, lack of neoadjuvant or adjuvant therapy for the primary tumour, palliative resection of the primary tumour, and any contraindications to liver transplantation. LITORALE’s inclusion and exclusion criteria are summarised in Table 1.

All patients received lymphadenectomy of the hepatic hilum and along the main hepatic artery at the time of LT or within one month before LT. No patient revealed lymph node metastases, which would have implied abortion of LT.

At present and in our national context, when patients with uCRLM fulfil criteria for LT within an approved and recognised protocol, they gain priority as macro-region urgency, providing that the proportion of patients with uCRLM does not exceed 5% of the total number of LTs performed in the same centre the year before [15].

The surgical team of the Department of Hepatobiliary and Transplant Surgery-Policlinico Sant’Orsola carried out all organ retrievals and transplant procedures, as well as the post-operative management of all recipients.

Immunosuppression was administered according to the standardised protocol of our centre, based on the use of corticosteroids and tacrolimus, as previously described [16]. Steroids were gradually reduced and stopped within six months, while tacrolimus trough levels were maintained between 8–12 ng/mL during the first four months post-transplant, and subsequently between 6–10 ng/mL. As part of the present protocol, an mTOR inhibitor (everolimus), was introduced as early as possible after one month from LT, contingent upon the patient’s clinical condition and laboratory findings.

Adjuvant chemotherapy was not formally incorporated into the post-transplant treatment protocol. Its use was instead evaluated on a case-by-case basis, considering the patient’s general condition, response to pre-transplant therapy, and the clinical judgment of the treating oncologist. When indicated, chemotherapy was started one month after transplantation and continued for at least six months, unless limited by toxicity or intolerance.

### 2.2. Variables and Outcome Measures

Variables assessed included patient demographics, BMI, comorbidities, history of prior liver resection, primary tumour location, and tumour staging according to the American Joint Committee on Cancer (AJCC) TNM classification [17]. Synchronous colorectal liver metastases were defined as metastases diagnosed within six months from the diagnosis of the primary tumour. Tumour burden score (TBS) was calculated as described by Sasaki et al. [18]; additionally, the OSLO score, developed in the SECA I study, was calculated for each patient [9]. Extended criteria donors (ECD) were defined according to the study of Nickkholgh et al. [19] Radiological responses to neoadjuvant chemotherapy were evaluated according to RECIST 1.1 criteria, distinguishing between stable disease (SD) and partial response (PR) [20]. Surgical complications were classified according to Clavien-Dindo classification, considering major complications for those ≥ 3a [21].

### 2.3. Statistical Analysis

Qualitative variables were presented as absolute numbers and percentages, while quantitative variables were expressed as median and interquartile range (IQR). Univariate analyses were conducted using Pearson’s chi-squared test or Fisher’s exact test for categorical variables, depending on sample size, and a Mann–Whitney U test for continuous variables. Survival analysis was conducted using the Kaplan–Meier estimators, and survival curves were compared through log-rank test. Hazard ratios (HR) with corresponding 95% confidence intervals (CI) were calculated using univariate Cox proportional hazards regression models. Two-sided *p*-values < 0.05 were considered statistically significant. Statistical analysis was performed using STATA version 18 (StataCorp LLC, College Station, TX, USA).

## 3. Results

The study included 21 patients, 8 in the pre-LITORALE group and 13 in the LITORALE group. Five patients in pre-LITORALE and twelve patients in LITORALE group were referred to our centre for evaluation for LT by other centres. The median age was significantly higher in the LITORALE group (60 years vs. 48 years, *p* = 0.033). Gender distribution across groups differed significantly: all pre-LITORALE patients were males, while females represented 53.8% of the LITORALE cohort (*p* = 0.011).

Liver resection before LT was more frequently performed in the LITORALE group (53.8% vs. 25%, *p* = 0.195). Interestingly, the patients in the pre-LITORALE cohort were all wild-type KRAS, whereas 46.2% of patients in the LITORALE cohort were KRAS-mutated (*p* = 0.032). Both cohorts included patients with BRAF wild-type primary tumour, synchronous CRLM, and who had undergone neoadjuvant therapy prior to LT. Neoadjuvant treatment had a median duration of 42.4 weeks in the pre-LITORALE group and 31.4 weeks after protocol introduction (*p* = 0.942). Regarding response according to RECIST criteria, stable disease was observed in 87.5% of the pre-LITORALE group and 53.8% of the LITORALE group, and partial response was observed in 12.5% and 46.2% of patients, respectively (*p* = 0.112). One patient in the pre-LITORALE group had extrahepatic disease, while all patients in the LITORALE had no extrahepatic disease, as per the stated eligibility criteria (*p* = 0.191).

The pre-LITORALE group had a higher median number of lesions (17.5 vs. 4, *p* = 0.004), larger major lesion size (5.5 cm vs. 3 cm, *p* = 0.082), and significantly higher TBS (18.02 vs. 6.32, *p* = 0.002); also, 50% of pre-LITORALE patients had lesions ≥ 5.5 cm, compared to 15.4% in the LITORALE group (*p* = 0.088). CEA levels were not significantly different amongst groups, although 25% of patients in the pre-LITORALE group had a CEA level above the limit of eligibility for the LITORALE protocol (≥80 μg/L, *p* = 0.058). OSLO scores were comparable amongst groups; notably, two patients in the pre-LITORALE group had an OSLO score = 2, compared to none in the LITORALE group (*p* = 0.164). The demographical results are summarised in Table 2.

LT waiting time was longer in the pre-LITORALE group (median 83.5 vs. 34 days, *p* = 0.346). The proportions of donors after neurological determination of death (DND), donors after cardiovascular determination of death (DCD), extended criteria donors (ECD) and living donors were similar in both groups (*p* = 0.242). It should be noted that in the pre-LITORALE group, two patients underwent LDLT (25% vs. 0%), while in the LITORALE group, three patients received a DCD donor graft, compared to one in the pre-LITORALE group (23.1% vs. 12.5%). HOPE was performed exclusively in the LITORALE group, with 84.6% of patients undergoing the procedure, (*p* < 0.001) with 134 min [IQR 112.5–190] of median duration. Veno-venous bypass was used in 25% of patients in the pre-LITORALE group and 15.4% in the LITORALE group (*p* = 0.586). Regarding caval reconstruction techniques, the piggyback technique was the most frequently adopted in both groups (75% in pre-LITORALE vs. 84.6% in LITORALE). Conventional reconstruction was used only in the LITORALE group (15.4%), while latero-lateral anastomosis and RAVAS were each performed in 12.5% of pre-LITORALE cohort but were not used in the LITORALE group (*p* = 0.209).

One patient in the LITORALE group died within 90 days of transplant due to sepsis complicated by multi-organ failure on the fifteenth day after LT.

Adjuvant chemotherapy after LT was administered to 87.5% of pre-LITORALE and 76.9% of LITORALE patients (*p* = 0.549). Disease recurrence occurred in 87.5% of pre-LITORALE patients and 46.2% of the LITORALE group (*p* = 0.058). The clinical results are summarised in Table 3.

The pattern of recurrence differed significantly amongst groups, with multi-site recurrence being the most frequent pattern in the pre-LITORALE group (50%), while accounting for only 7.7% of the LITORALE group (*p* = 0.048). Also, lung-only recurrences occurred only in the LITORALE group (50%, *p* = 0.033). Table 4 summarises the patients who developed recurrence after LT, and the therapeutical strategies employed to treat the recurrence.

**Figure 1:**
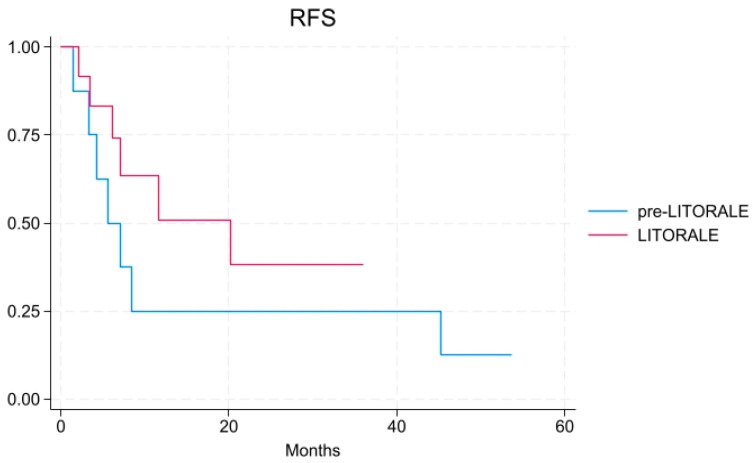
Recurrence-free survival (RFS) in the LITORALE and pre-LITORALE group [HR 0.54, 95% CI: 0.17–1.69].

**Figure 2:**
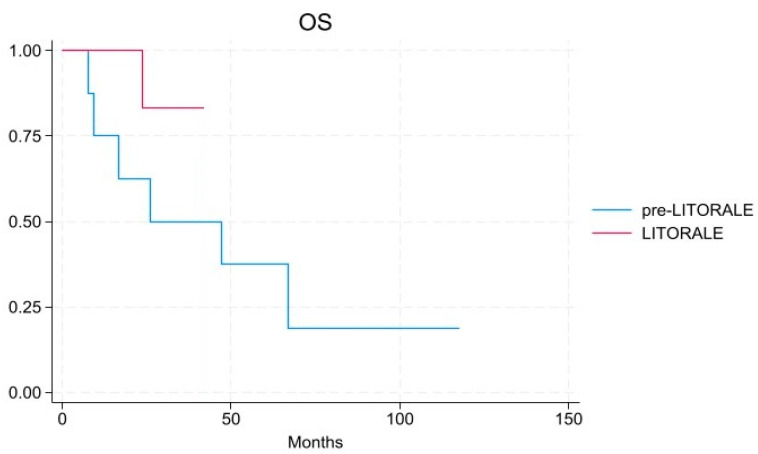
Overall survival in the LITORALE and pre-LITORALE group [HR 0.52, 95% CI: 0.09–2.97].

Concerning survival rates, median recurrence-free survival (RFS) was 6.4 months in pre-LITORALE and 7.8 months in LITORALE (*p* = 0.589); median OS was 36.5 months in pre-LITORALE and 22.5 months in the LITORALE group, respectively (*p* = 0.189) (Figure 1 and Figure 2). One- and three-year OS rates were 75% and 50% in pre-LITORALE vs. 100% and 83% in LITORALE (*p* = 0.456).

## 4. Discussion

Liver transplantation represents a novel and promising option for the treatment of uCRLM, whose prognosis would otherwise be poor due to the limited therapeutical tools available. The recent evidence on this practice has shown a progressive increase in survival rates through a meticulous refinement of transplant eligibility criteria. The primigenial SECA I trial demonstrated a 60% 5-year OS, although it was burdened with a high recurrence rate [9]; on the other hand, the SECA II trial reached a 5-year OS of 83% as a result of improved patient selection criteria, which encompassed fewer metastases, lower TBS, and lower serum CEA levels. Not only did those patients have higher presumptive survival according to prognostic indexes (such as Fong Clinical Risk Score and Oslo score), but they also had lower FDG-PET uptake values compared to the patients in the SECA I [10]. The impact of refining eligibility criteria on post-transplant outcomes is clear also from further large series published on the topic, such as the TransMet randomised clinical trial and the University of Rochester Medical Center prospective trial, whose survival rates reached, and in some cases, outperformed the ones observed after transplantation for other hepatic malignancies [11,12].

In this study, we evaluated how the introduction of standardised criteria for transplant eligibility (LITORALE protocol) impacted on liver transplantation outcomes in uCRLM patients and reported our preliminary findings. The results showed that the introduction of standardised selection criteria influenced the extent of disease for which patients were considered for transplantation, with a lower TBS, number, and maximum size of lesions in patients transplanted after protocol’s inception. As per the protocol’s inclusion criteria, none of the patients in the LITORALE group had serum CEA levels ≥ 80 μg/L or extrahepatic disease; moreover, although not statistically significant, the percentage of patients with partial response to treatment according to mRECIST increased after the standardization of eligibility criteria (46.2% vs. 12.5%).

Adjuvant chemotherapy was widely adopted in our study population, with 87.5% of patients in the pre-LITORALE group and 76.9% in the LITORALE group receiving post-LT treatment. This widespread use confirms that systemic therapy after LT is not only feasible but routinely implemented in selected patients, even under immunosuppressive regimens [22]. These data support the rationale for considering adjuvant chemotherapy in patients undergoing liver transplantation. Importantly, the LITORALE cohort showed a 3-year OS of 83%, which compares favourably to the 3-year OS of only 26.6% observed in patients treated with chemotherapy alone in the randomised TransMet trial [11]. This highlights the potential survival benefit of transplantation-based strategies in highly selected cases.

The use of immune checkpoint inhibitors (ICIs) in patients undergoing liver transplantation is a developing area of clinical interest. In the pre-transplant setting, ICIs may offer a strategy to downstage tumours and expand transplant eligibility. However, their use has been associated with a significant risk of acute rejection after transplantation, even when administered months before surgery [23,24]. Similarly, using ICIs to treat recurrent disease post-transplant poses challenges, given the fine balance between stimulating anti-tumour immunity and preserving graft tolerance. Some data suggest that waiting at least 3–6 months post-ICI therapy before proceeding to transplant may reduce rejection risk [25]; however, additional prospective studies are required to verify this approach.

Although survival rates in our study population resulted comparable, patients transplanted within the LITORALE protocol showed different recurrence patterns, with a lower rate of multi-site recurrences (7.7% vs. 50%, *p* = 0.048) and a higher rate of lung-only recurrences (50% vs. 0, *p* = 0.033); this result is clinically relevant, as lung-only recurrences have been associated with a better prognosis compared to other sites in a recent study on tumour recurrence after LT for uCRLM from the Oslo group [26].

Our survival results were comparable to the SECA II trial, with a 100% 1-year OS and an 83% 3-year OS [10]; similarly to the SECA-II trial, we refined our criteria for transplant eligibility, enrolling patients with lower liver tumour burden, better tumour response to treatment and lower serum CEA levels. This strategy is likely to have led to more favourable recurrence patterns in the cohort of patients transplanted within the LITORALE protocol, although the limited number of patients and the short study timespan advocate future validation.

As remarked in other publications on the topic, a multidisciplinary approach is a factor of paramount importance to direct the diagnostic and therapeutical pathway of patients with CRLM [27,28]. In our experience, multidisciplinary screening for transplant eligibility allowed a more precise evaluation of patients and contributed to the outcome modifications, similarly to what is reported in the TransMet and University of Rochester Medical Center trials [11,12].

The persistent shortage of donors remains a major limitation in implementing LT for uCRLM on a large scale. While LDLT represents a viable strategy [29,30]—offering favourable outcomes with no influence on deceased donor waiting lists—living donation is not always available or feasible; in this sense, ECD and DCD grafts offer valid alternatives to expand the donor pool [31,32,33,34]. We employed a higher number of ECD (6, 46.2%) and DCD (3, 23.1%) graft in the group of patients transplanted after protocol’s inception, whose functional and survival outcomes resulted comparable; indeed, uCRLM patients can be favourably matched with marginal donors due to preserved liver function and the absence of portal hypertension. These findings reinforce the growing evidence that, when managed with appropriate reconditioning, DCD and ECD grafts can achieve satisfactory functional and survival outcomes after transplant [34,35,36]. Two-stage procedures with split LT and delayed hepatectomy—such as RAPID and RAVAS—also represent a promising strategy to enlarge the donor pool for uCRLM patients without interfering with deceased donor waiting lists [37,38,39].

## 5. Limits

This study has some limitations. First, the limited size of the study may affect the capability to identify subtle but clinically relevant differences between the groups. Moreover, the short timespan of the LITORALE protocol implied a limited follow-up for the latest transplanted patients, although a minimum follow-up of six months was provided for all patients. Future studies on a larger number of patients with longer follow-up are needed to further infer the clinical effects of implementing string eligibility criteria in LT for uCRLM. Also, future transplant eligibility criteria will likely have to include novel biomarkers and imaging techniques to better refine patient selection and achieve even more favourable outcomes.

## 6. Conclusions

In conclusion, our experience aligns with recently emerging evidence in showing that a structured, multidisciplinary approach with refined selection criteria can significantly influence survival and oncological outcomes of LT for uCRLM. While still preliminary, our results support the continuous development of tailored protocols such as LITORALE, whose clinical impact is expected to become more and more relevant as access to LT becomes more available for novel oncological indications.

## Figures and Tables

**Table 1:** LITORALE protocol inclusion and exclusion criteria.

**Inclusion Criteria**
Age between 18 and 73 years.
Histologically confirmed colorectal adenocarcinoma previously treated with curative intent (pT4a, R0 resection).
uCRLM at diagnosis or due to recurrence after previous liver resection.
No signs of recurrence of the primary (PET, CT, colonoscopy).
No evidence of extrahepatic disease (PET and/or CT).
ECOG 0-1
Neutrophils > 1.0 × 10^9^/L (without G-CSF).
At least one line of chemotherapy, with SD or PR (mRECIST) for at least 3 months.
CEA < 80 µg/L, or ≥50% decrease from the highest previous CEA level.
Written informed consent and expected patient cooperation for treatment and follow-up.
No contraindications to liver transplant per institutional protocol.
**Exclusion Criteria**
Presence of other neoplasms.
Local recurrence of the primary tumour.
Presence of extrahepatic metastatic disease.
Patients not treated with neoadjuvant or adjuvant conventional therapy for the primary tumour.
Palliative resection of the primary tumour.

**Table 2:** Patients’ demographic and disease characteristics.

Variables	Study Population (n = 21)	Pre-LITORALE (n = 8)	LITORALE (n = 13)	*p*-Value
Age in years, median [IQR]	53 [48–61]	48 [43.1–54.5]	60 [51–63.7]	**0.033**
Gender				**0.011**
Female, n (%)	7 (33.3)	0	7 (53.8)	
Male, n (%)	14 (66.7)	8 (100)	6 (46.2)	
BMI in kg/m^2^, median [IQR]	23.4 [21.6–27.8]	22.8 [21.9–25.15]	24.5 [21.6–28]	0.447
Previous liver resection, n (%)	9 (42.9)	2 (25)	7 (53.8)	0.195
Primary tumour site				0.675
Right, n (%)	3 (14.3)	1 (12.5)	2 (15.4)	
Left, n (%)	10 (47.6)	3 (37.5)	7 (53.8)	
Rectum, n (%)	8 (38.1)	4 (50)	4 (30.8)	
(y)pT stage				0.701
0, n (%)	1 (4.8)	0	1 (7.7)	
1, n (%)	0	0	0	
2, n (%)	2 (9.5)	1 (12.5)	1 (7.7)	
3, n (%)	17 (81.0)	7 (87.5)	10 (76.9)	
4a, n (%)	1 (4.8)	0	1 (7.7)	
(y)pN stage				0.586
0, n (%)	5 (23.8)	1 (12.5)	4 (30.8)	
1, n (%)	10 (47.6)	4 (50)	6 (46.2)	
2, n (%)	6 (28.6)	3 (37.5)	3 (23.1)	
KRAS				**0.023**
wt, n (%)	14 (66.7)	8 (100)	7 (53.8)	
mt, n (%)	6 (33.3)	0	6 (46.2)	
BRAF				-
wt, n (%)	21 (100)	8 (100)	13 (100)	
mt, n (%)	0	0	0	
Synchronous CRLM, n (%)	21 (100)	8 (100)	13 (100)	-
Neoadjuvant therapy prior to LT, n (%)	21 (100)	8 (100)	13 (100)	-
Extrahepatic disease, n (%)	1 (4.8)	1 (12.5)	0	0.191
mRECIST				0.112
SD, n (%)	14 (66.7)	7 (87.5)	7 (53.8)	
PR, n (%)	7 (33.3)	1 (12.5)	6 (46.2)	
n. of metastases at diagnosis, n (%)				**0.011**
≤5	7 (33.3)	0	7 (53.8)	
5 < x ≤ 10	6 (28.6)	2 (25.0)	4 (30.8)	
>10	8 (38.1)	6 (75.0)	2 (15.4)	
Largest lesion size at diagnosis in cm, median [IQR]	4 [2.5–6]	5.5 [3.65–8.8]	3 [1.8–4.2]	0.082
Tumour burden score, median [IQR]	9.03 [5.72–15.65]	18.02 [13.13–24.97]	6.32 [3.16–9.03]	**0.002**
Oslo score, n (%)				0.164
0, n (%)	10 (47.6)	3 (37.5)	7 (53.8)	
1, n (%)	9 (42.9)	3 (37.5)	6 (46.2)	
2, n (%)	2 (9.5)	2 (25)	0	
CEA in µg/L, median [IQR]	8.7 [2.8–17.6]	12.5 [3.1–50.55]	4.6 [2.8–13.6]	0.365
CEA ≥ 80 µg/L, n(%)	2 (9.5)	2 (25)	0	0.058

**Table 3:** Post-transplant outcomes.

Variables	Study Population (n = 21)	Pre-LITORALE (n = 8)	LITORALE (n = 13)	*p*-Value
LT waiting time in days, median [IQR]	37 [11–78]	83.5 [14–176.5]	34 [11–71]	0.346
Interval from primary resection and LT in days, median [IQR]	389 [331–892]	382.5 [231.5–522]	389 [337–1032]	0.192
Donor type				0.242
DBD, n (%)	7 (33.3)	3 (37.5)	4 (30.8)	
EC-DBD, n (%)	8 (38.1)	2 (25)	6 (46.2)	
DCD, n (%)	4 (19)	1 (12.5)	3 (23.1)	
LD, n (%)	2 (9.5)	2 (25)	0	
HOPE, n (%)	11 (52.4)	0	11 (84.6)	**<0.001**
HOPE duration in minutes, median [IQR]		0	134 [112.5–190]	-
Veno-venous bypass, n (%)	4 (19)	2 (25)	2 (15.4)	0.586
Caval reconstruction, n (%)				0.209
Conventional	2 (9.5)	0	2 (15.4)	
Piggyback	17 (81)	6 (75)	11 (84.6)	
Latero-lateral	1 (4.8)	1 (12.5)	0	
RAVAS	1 (4.8)	1 (12.5)	0	
n°. of lesions at pathology, n (%)				0.546
≤5	6 (28.6)	2 (25)	4 (30.8)	
5 < x ≤ 10	3 (14.3)	2 (25)	1 (7.7)	
>10	12 (57.1)	4 (50)	8 (61.5)	
Largest lesion size at pathology, cm, median [IQR]	3.5 [1.55–7.8]	5.45 [2.75–9.25]	2.75 [1.2–6.35]	0.203
Clavien–Dindo ≥ 3a, n (%)	3 (14.3)	2 (25)	1 (7.7)	0.271
ICU stay, d, median [IQR]	3 [2–5]	4 [3–5]	3 [2–4]	0.205
LOS, d, median [IQR]	12 [10–20]	15 [11.5–26.5]	10 [10–12]	0.096
90-days mortality, n (%)	1 (4.8)	0	1 (7.7)	0.646
LT-adjuvant CHT, n (%)	17 (81)	7 (87.5)	10 (76.9)	0.549
Recurrence, n (%)	13 (61.9)	7 (87.5)	6 (46.2)	0.058
Pattern of recurrence				**0.048**
No recurrence, n (%)	8 (38.1)	1 (12.5)	7 (53.8)	
Single-site, n (%)	8 (38.1)	3 (37.5)	5 (38.5)	
Multi-site, n (%)	5 (23.1)	4 (50)	1 (7.7)	
Site of recurrence				
Liver-only, n (%)	2/13 (15.4)	1/7 (14.3)	1/6 (16.7)	0.906
Lung-only, n (%)	3/13 (23.1)	0	3/6 (50)	**0.033**
Lymph nodes-only, n (%)	1/13 (7.7)	1/7 (14.3)	0	0.335
Bone-only, n (%)	1/13 (7.7)	1/7 (14.3)	0	0.335
Pelvic, n (%)	1/13 (7.7)	0	1/6 (16.7)	0.261
Multiorgan, n (%)	5/13 (38.4)	4/7 (57.1)	1/6 (16.7)	0.135

**Table 4:** Characteristics of patients’ recurrences and their respective treatment.

Patient	LITORALE	Site of Recurrence	Treatment of Recurrence			
			Chemiotherapy	Radiotherapy	Surgery	Type of Surgery
1	0	Abdominal lymph node	Yes		Yes	Lymph node resection
2	0	Liver				
3	0	Lung + brain	Yes	Yes	Yes	Lung resection
4	0	Lung + liver + adrenal gland	Yes	Yes	Yes	Lung resection
5	0	Bone				
6	0	Lung + liver			Yes	Lung resection
7	0	Lung + brain + liver + kidney + bone	Yes			
8	1	Pelvic			Yes	Abdominoperineal rectal resection
9	1	Lung + bone	Yes	Yes		
10	1	Liver			Yes	Liver resection
11	1	Lung	Yes	Yes		
12	1	Lung	Yes	Yes		
13	1	Lung	Yes			

## Data Availability

The data that support the findings of this study are available from the corresponding author upon reasonable request.

## Abbreviations

The following abbreviations are used in this manuscript:

BMI	Body mass index
CEA	Carcinoembryonic antigen
CI	Confidence intervals
CRC	Colorectal cancer
CRLM	Colorectal liver metastases
DCD	Donors after cardiovascular determination of death
DND	Donors after neurological determination of death
ECD	Extended criteria donors
ECOG	Eastern cooperative oncology group
HR	Hazard ratio
ICIs	Immune checkpoint inhibitor
IQR	Interquartile range
LDLT	Living-donor liver transplantation
LT	Liver transplantation
OS	Overall survival
PET	Positron emission tomography
PR	Partial response
RECIST	Response evaluation criteria in solid tumours
SD	Stable disease
TBS	Tumour burden score
uCRLM	Unresectable colorectal liver metastases
